# The Global Burden of Absenteeism Related to COVID-19 Vaccine Side Effects Among Healthcare Workers: A Systematic Review and Meta-Analysis

**DOI:** 10.3390/vaccines12101196

**Published:** 2024-10-19

**Authors:** Marios Politis, Georgios Rachiotis, Varvara A. Mouchtouri, Christos Hadjichristodoulou

**Affiliations:** 1 Department of Hygiene and Epidemiology, School of Medicine, University of Thessaly, 41222 Larissa, Greece; 2 Center for Epidemiology and Community Medicine, Stockholm Health Care Services, 10431 Stockholm, Sweden

**Keywords:** absenteeism, healthcare workers, COVID-19 vaccines, vaccine side effects

## Abstract

**Background:** A rise in absenteeism among healthcare workers (HCWs) was recorded during the COVID-19 pandemic, mostly attributed to SARS-CoV-2 infections. However, evidence suggests that COVID-19 vaccine-related side effects may have also contributed to absenteeism during this period. This study aimed to synthesize the evidence on the prevalence of absenteeism related to COVID-19 vaccine side effects among HCWs. **Methods:** The inclusion criteria for this review were original quantitative studies of any design, written in English, that addressed absenteeism related to the side effects of COVID-19 vaccines among HCWs. Four databases (PubMed, Scopus, Embase, and the Web of Science) were searched for eligible articles on 7 June 2024. The risk of bias was assessed using the Newcastle–Ottawa scale. Narrative synthesis and a meta-analysis were used to synthesize the evidence. **Results:** Nineteen observational studies with 96,786 participants were included. The pooled prevalence of absenteeism related to COVID-19 vaccine side effects was 17% (95% CI: 13–20%), while 83% (95% CI: 80–87%) of the vaccination events did not lead in any absenteeism. Study design, sex, vaccination dose, region, and vaccine type were identified as significant sources of heterogeneity. **Conclusions:** A non-negligible proportion of HCWs were absent from work after reporting side effects of the COVID-19 vaccine. Various demographic factors should be considered in future vaccination schedules for HCWs to potentially decrease the burden of absenteeism related to vaccine side effects. As most studies included self-reported questionnaire data, our results may be limited due to a recall bias. **Other:** The protocol of the study was preregistered in the PROSPERO database (CRD42024552517).

## 1. Introduction

The COVID-19 pandemic imposed significant pressure on healthcare systems worldwide [1,2,3,4]. Common issues included limited access to and the decreased utilization of healthcare services, the inadequate provision of healthcare services, unexpected changes in service delivery, staff shortages, and a lack of necessary equipment [1,3,5,6,7,8].

A rise in absenteeism in healthcare workers (HCWs) has been described in terms of both frequency and duration during the COVID-19 pandemic [9,10,11]. Although absenteeism in HCWs during this period was mostly attributed to SARS-CoV-2 infections, an increase in absenteeism for reasons unrelated to SARS-CoV-2 infections, such as strict infection control measures for suspected COVID-19 cases or burnout, was also observed [9,10,11,12]. Evidence from cross-sectional and cohort studies using self-reported questionnaire data and electronic records suggests that side effects related to COVID-19 vaccines contributed to absenteeism among HCWs during the pandemic [12,13,14,15]. Sociodemographic factors, such as occupation and sex, as well as vaccine-related factors, like dose and the type of vaccine, have been found to be associated with absenteeism among HCWs [14,15].

A vaccine side effect is considered any health problem that is related to the vaccination and can be caused by any vaccine type [16,17]. Most vaccine side effects are common among different vaccine types and are mainly the result of reactogenicity, the physical manifestation of the inflammatory response to a vaccination event [16,18]. Common side effects of vaccines, including COVID-19 vaccines, are pain and redness at the injection site, fever, fatigue, and muscle pains [16,17,19].

Sick-related absenteeism in HCWs has been associated with a decreased quality of care and increased costs for patients, as well as unplanned shifts, a stressful working environment, and a higher likelihood of medical errors due to staff shortages [9,20,21,22]. Moreover, sick-related absenteeism in HCWs may contribute to a vicious cycle, wherein staff shortages lead to additional work absences, perpetuating the problem of absenteeism [23].

Our aim was to systematically synthesize the latest evidence to explore the association between COVID-19 vaccination side effects and work absence in HCWs, addressing the following research questions:

What percentage of the COVID-19 vaccination events led to absenteeism that was associated with vaccine-related side effects among HCWs?

What factors were associated with the absenteeism that was related to COVID-19 vaccine-related side effects among HCWs?

What was the duration of absenteeism that was related to COVID-19 vaccine-related side effects among HCWs?

## 2. Materials and Methods

The current systematic review was reported according to the latest PRISMA guidelines [24]. A protocol of the study was uploaded to the PROSPERO database before the initial screening of the evidence (CRD42024552517).

### 2.1. Inclusion Criteria and Search String

According to the Population, Intervention, Comparison, Outcomes, and Study (PICOS) framework, the inclusion criteria for the current systematic review were original quantitative studies of any design written in English (S), addressing sick-related absenteeism (O) that was related to the side effects of COVID-19 vaccines (I) among healthcare workers (P). As our study focused on the prevalence of sick-related absenteeism, no comparison group needed to be defined.

Based on the inclusion criteria, a search string was created first in PubMed and then adapted for use in Scopus, Embase, and the Web of Science using the themes (COVID-19 vaccine) AND (absenteeism) AND (healthcare workers). The detailed search strings can be found in the Supplementary Materials.

### 2.2. Screening of the Evidence, Quality Assessment, and Data Extraction

The title/abstract screening and the full-text screening were conducted independently by two researchers (M.P. and G.R.). Disagreements between the researchers at each step were resolved through consensus.

The quality assessment of the included studies was performed using the Newcastle–Ottawa assessment tool for cross-sectional and cohort studies. Each researcher (M.P. and G.R.) independently conducted the assessment, and any differences were resolved through discussion to reach consensus. Studies of satisfactory quality were considered to be cross-sectional studies with a score of 5 or more or cohort/prospective studies with a score of 4 or more in all domains.

Data extraction was conducted by M.P. and subsequently validated by G.R. The extracted data included the first authors, year of publication, country, study design, population characteristics, raw data and the percentages of absenteeism related to COVID-19 vaccine side effects, factors associated with absenteeism related to COVID-19 vaccine side effects, and data regarding the duration of absenteeism (mean days and median days).

### 2.3. Evidence Synthesis

All the statistical analyses were performed with the STATA BE 18 version (StataCorp, College Station, TX, USA). A random-effects, inverse-variance model was used to estimate the pooled proportions with 95% confidence intervals (95% CI) and the 95% prediction interval synthesizing all the available evidence regarding the outcome: the prevalence of at least one day of absenteeism related to COVID-19 vaccine side effects among HCWs vaccinated with a COVID-19 vaccine. The random-effects model was chosen due to the observed between-study heterogeneity. The between-study heterogeneity was assessed using the I^2^ statistic and the 95% prediction interval. To capture the total burden of COVID-19 vaccination on sick-related absenteeism, we treated serial vaccinations in the same individuals as independent events in our main analysis. All types of COVID-19 vaccines, different vaccine combinations, and various vaccine doses were included in the main analysis. The publication bias was assessed both qualitatively and quantitatively using the DOI and LFK index, as well as the funnel plot and the Egger’s test. A narrative synthesis of the evidence was used when the data were not compatible with the meta-analysis, such as for factors associated with absenteeism related to COVID-19 vaccine side effects (other than those included in the subgroup analysis) and the duration of absenteeism.

The subgroup analysis was conducted only when each subgroup included three or more studies. Subgroup analyses for sex, vaccine type, region, and study design were performed using the random-effects, inverse-variance meta-analysis model to explore the potential sources of heterogeneity. In the by-region subgroup analysis, a single study from Africa was excluded, and three regional groups were considered: Europe, USA, and Asia. For the vaccination dose subgroup analysis, we conducted a restricted maximum likelihood, random-effects, bivariate meta-analysis to account for the data dependency. Since individual data on the presence of absenteeism among HCWs after the first and second COVID-19 vaccine doses were lacking, we assumed no correlation between the presence of absenteeism and the vaccination dose. Additionally, we conducted two sensitivity analyses, assuming moderate positive and negative correlations (±0.5) for the presence of absenteeism after the first and second COVID-19 vaccine doses. The between-study heterogeneity in the latter subgroup analysis was assessed using the Jackson–White–Riley I^2^ statistic. The Cochrane Q statistic was used to identify differences between the subgroups. Last, the random-effects, inverse-variance meta-analysis model was used in the post-hoc sensitivity analysis to examine whether the study design contributed to the between-group heterogeneity among the recipients of the two different types of mRNA COVID-19 vaccines.

## 3. Results

### 3.1. Flow Chart

**Figure 1:**
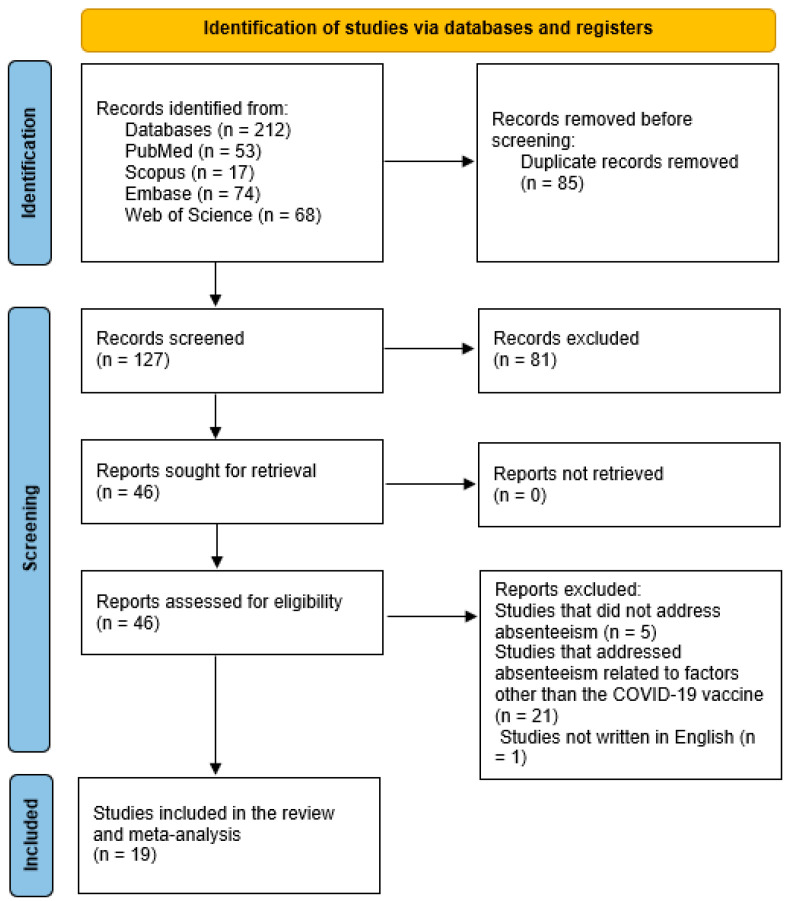
Flow chart of the records/articles identified, screened, and included for evidence synthesis.

Our search in all the databases was conducted on 7 June 2024. In total, 212 results were retrieved, and 127 were further screened after the exclusion of 85 duplicates (Figure 1). After the title/abstract screening, 46 articles were eligible for the full-text screening. After the exclusion of 27 articles for specific reasons, 19 articles were included in this review [14,15,25,26,27,28,29,30,31,32,33,34,35,36,37,38,39,40,41]. Specific reasons for exclusion included studies that did not address absenteeism, studies that addressed absenteeism related to factors other than COVID-19 vaccine side effects, and studies not conducted in English.

### 3.2. Main Characteristics of the Included Studies and Quality Assessment

In total, 14 cross-sectional studies and 5 cohort/prospective studies were considered for the evidence synthesis, including 96,786 HCWs from secondary or tertiary care (Table 1). Out of the 14 cross-sectional studies and 5 cohort/prospective studies, 9 and 4, respectively, were assessed as having satisfactory quality. A common issue among the cross-sectional studies was the lack of description of the non-responders. The majority of both the cross-sectional and the cohort/prospective studies primarily relied on self-reported questionnaire information regarding the outcome of interest.

### 3.3. Prevalence of at Least One Day of Absenteeism Related to COVID-19 Vaccine Side Effects Among HCWs

**Figure 2:**
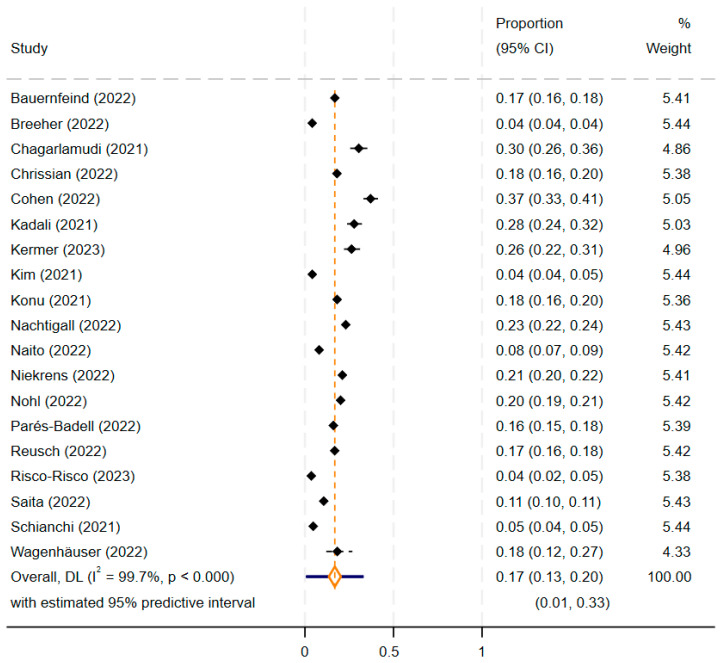
Forest plot of the meta-analysis, depicting the prevalence of absenteeism of at least one day among healthcare workers, related to COVID-19 vaccine side effects overall and by study design. The estimated prevalence for each individual study is presented with black diamonds, while the pooled estimated proportion is shown with an orange diamond. The blue line represents the 95% prediction interval [14,15,25,26,27,28,29,30,31,32,33,34,35,36,37,38,39,40,41].

A total of 17% (14,163/157,981; 95% CI: 13–20%) of the vaccination events led to at least one day of absenteeism related to COVID-19 vaccine side effects among HCWs (Figure 2), while 83% (95% CI: 80–87%) of the vaccination events did not lead in any absenteeism. A high heterogeneity between the included studies was observed (I^2^ = 99.7%, 95% prediction interval: 1–33%).

### 3.4. Subgroup Analysis

In the subgroup analysis, five significant sources of between-group heterogeneity were identified (Table 2). The pooled proportions significantly differed between the first vaccination (7%; 95% CI: 4–10%) and the second (20%; 95% CI: 14–26%) vaccination; the cross-sectional studies (19%; 95% CI: 14–23%) and the cohort/prospective studies (8%; 95% CI: 3–14%); the male participants (17%; 95% CI: 13–21%) and the female (26%; 95% CI: 20–32%) participants; and the mRNA-1273 vaccine (23%; 95% CI: 16–31%) and the BNT162b2 mRNA (12%; 95% CI: 9–15%) vaccine. In the by-region analysis, the studies from Asia recorded an 8% (95% CI: 4–12%) prevalence of absenteeism after a COVID-19 vaccination event, while the studies from Europe and the USA recorded a prevalence of 17% (95% CI: 11–22%) and 23% (95% CI: 11–35%), respectively. Still, the subgroup heterogeneity remained high, with the I^2^ ranging from 82.8% to 99.9%. In the sensitivity analysis, the prevalence of absenteeism between the first and second doses was significantly different, even when considering the different levels of correlation regarding the presence of absenteeism after the first and second COVID-19 vaccine doses.

### 3.5. Risk of Publication Bias Assessment

**Figure 3:**
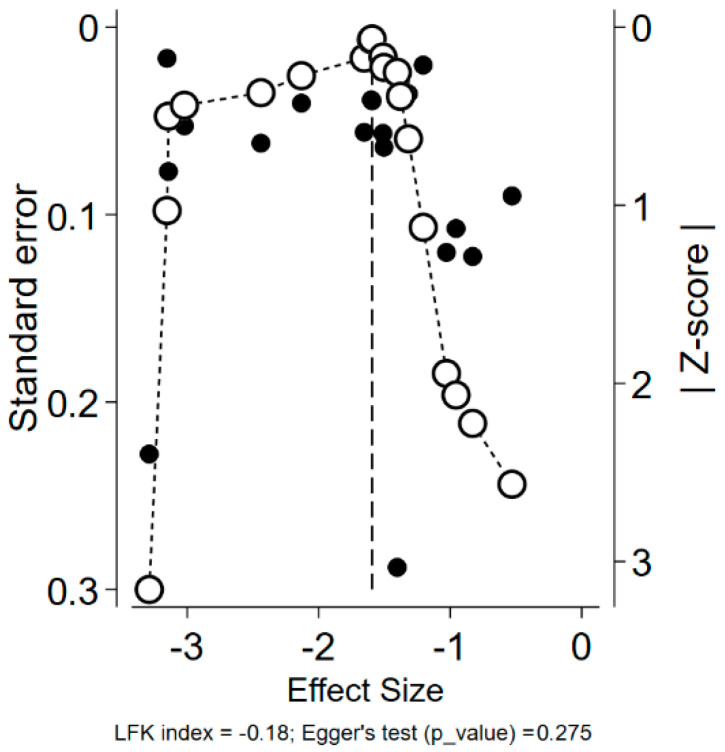
Funnel and DOI plots along with the Egger’s test and the LFK index, assessing the risk of a publication bias in the meta-analysis.

When visually assessing the DOI and funnel plot, we considered the risk of a publication bias in our main analysis to be very low, as no considerable asymmetry was observed. The qualitative assessment is supported by objective assessments, as both the LFK index (0.18) and the Egger’s test (*p*-value = 0.275) did not identify any significant asymmetry (Figure 3).

### 3.6. Other Factors Associated with at Least One Day of Absenteeism Related to COVID-19 Vaccine Side Effects Among HCWs

A younger age, especially of below 55 years old, was positively associated with reporting at least one day of sick leave [15,25,26,28,35]. Two studies reported data on the prevalence of absenteeism after the third COVID-19 vaccination, with rates of 8% and 27.9% [14,34]. In the first study, the prevalence of absenteeism was not significantly different between the second and third COVID-19 vaccine doses [34]. However, in the second study, the prevalence of absenteeism differed significantly between the second and third COVID-19 vaccine doses, with a higher rate observed after the third dose [14]. The first-dose recipients of vector vaccines (compared to mRNA vaccines), the second-dose recipients of mRNA-1273 (compared to other mRNA or vector vaccines), and the recipients of heterologous vaccine combinations (compared to homologous vaccine combinations) were found to be more likely to report at least one day of absenteeism related to COVID-19 vaccine side effects [14,15,25,28,32,34,36,37,41]. Physicians and HCWs who were directly involved in patient care were less likely to report at least one day of absenteeism, compared to nurses and HCWs with no direct patient contact, respectively [14,25,26,28,29]. Lastly, other factors associated with absenteeism related to COVID-19 vaccine side effects included the number of household members, income, race, private vs. academic department, absenteeism during the first dose, and the history of chronic illness or allergies [28,29,37].

### 3.7. Duration of Absenteeism Related to COVID-19 Vaccine Side Effects Among HCWs Who Reported a Sick Leave

The vast majority of the participants reported one or two days of work absence, with only a few needing three or more days (Table 3). No considerable differences in the duration of sick leave were observed between the first, second, and third dose.

## 4. Discussion

After a systematic literature review, we synthesized the latest evidence from 19 observational studies. We reported that 17% of the total vaccination events led to at least one day of absenteeism related to COVID-19 vaccine side effects among HCWs. We reported that female sex (compared to male), the mRNA-1273 vaccine (compared to the BNT162b2 mRNA vaccine), and the second vaccination (compared to the first) were positively associated with at least one day of absenteeism related to COVID-19 vaccine side effects among HCWs. We also reported that the prevalence of absenteeism related to COVID-19 vaccine side effects differed between regions among HCWs (Europe, Asia, and the USA). A younger age, heterologous vaccine combinations, and being a physician or being directly involved in patient care were also identified as factors positively associated with reporting at least one day of absenteeism related to COVID-19 vaccine side effects among HCWs. Among those who reported sick leave, the vast majority reported taking one or two days off work.

HCWs were a vaccination priority group during the COVID-19 pandemic [42]. Moreover, being vaccinated against infectious diseases is considered a moral obligation and a part of good medical practice among HCWs, and in many cases, COVID-19 vaccination was mandatory for HCWs during the pandemic [43,44,45]. These factors explain why a very high proportion of HCWs got vaccinated against COVID-19, reaching a global prevalence of 77.3% [46]. Given the relatively short vaccination dose interval for various types of COVID-19 vaccines (3 to 12 weeks), a prevalence of absenteeism related to COVID-19 vaccine side effects as high as 17% may have contributed to healthcare service disruptions during the pandemic [27,47,48]. As Chagarlamudi et al. described, their department confronted disruptions in care delivery due to absences from work caused by COVID-19 vaccine-related side effects [27]. Specifically, cancellations and delays in service provision were observed as many employees scheduled their vaccinations on the same day, resulting in simultaneous absences from work [27]. The authors further noted that during the days of increased absenteeism, patients experienced dissatisfaction with the services provided [27].

Considering the above, our findings provide valuable information for planning future vaccine administration schedules for HCWs, especially during emergencies such as a pandemic, given that the side effect profiles of the vaccines will be similar to those of the COVID-19 vaccines. First, a higher prevalence of absenteeism related to vaccine side effects is expected after the second vaccination compared to the first. Age and sex should be considered in vaccination planning, as the likelihood of reporting absenteeism due to COVID-19 vaccine side effects varied between men and women and between people aged <55 years and ≥55 years. We, therefore, propose three groups with different likelihoods of reporting absenteeism after a COVID-19 vaccination: higher for women younger than 55 years old, moderate for men younger than 55 years old and for women aged 55 years and older, and lower for men aged 55 years and older. Moreover, it should be noted that physicians and HCWs who were directly involved in direct patient care were the least likely occupational group to report at least one day of absenteeism. Lastly, the individual demographic composition of each department should be taken into consideration when planning a vaccination schedule within a healthcare unit.

It has been reported that a younger age, female sex, and receiving a second vaccination dose (compared to the first dose) were associated with increased reactogenicity and prevalence of side effects following a COVID-19 vaccination [14,15,49,50,51]. These factors may explain the differences in reported absenteeism among these subgroups in our analysis. The above explanation may also apply to the differences in reported absenteeism observed between heterologous and homologous COVID-19 vaccine combinations, as well as between the two mRNA vaccines (mRNA-1273 and BNT162b2 mRNA) [14,15,41,52,53]. However, other factors that were not directly related to immune response and reactogenicity, such as occupation group, income, or the number of household members, were also found to be associated with absenteeism after COVID-19 vaccination. These relationships should be further explored in the future to better understand their nature.

According to our by-region subgroup analysis, the prevalence of absenteeism related to COVID-19 vaccine side effects varied between Europe, the USA, and Asia. Individual, institutional, national, or regional factors may have contributed to these differences. Socio-cultural, health-related, and institutional regulatory factors have been previously described as related to absenteeism among both healthcare workers and other occupations [54,55,56,57,58,59,60,61]. Further research exploring factors at all levels during the COVID-19 pandemic is expected to provide valuable insights into our findings.

As previously discussed, absenteeism among HCWs during the COVID-19 pandemic was primarily attributed to SARS-CoV-2 infections [9,10,11]. Indeed, when compared to absenteeism related to SARS-CoV-2 infections, absenteeism due to COVID-19 vaccine side effects had a lower burden, both in terms of prevalence and duration [9]. However, an increase in all-cause absenteeism was observed among HCWs during the COVID-19 pandemic [11]. Our findings contribute to the existing literature by proposing that absenteeism related to COVID-19 vaccine side effects may have been an important component of the increased all-cause absenteeism during the pandemic.

Although the evidence on the relationship between absenteeism related to vaccine side effects among HCWs for vaccines other than COVID-19 is limited, absenteeism related to influenza vaccine side effects appears to be lower compared to that of the COVID-19 vaccine. Three cross-sectional studies, using self-reported data, reported a prevalence of absenteeism related to influenza vaccine side effects of 1–5% among HCWs. Specifically, 2% (24/1203) of participants from six UK hospitals vaccinated against influenza during the 2002/2003 season reported an absence from work [62]. Moreover, among influenza-vaccinated personnel at a tertiary children’s hospital during the 1989–90 influenza season, only 1% (4/333) reported a work absence [63]. Lastly, 5% (13/266) of hospital HCWs who participated in a survey regarding influenza vaccine side effects during the 1988–89 season reported missing work [64].

### Strengths and Limitations

To our knowledge, this is the first systematic review and meta-analysis reporting on absenteeism related to COVID-19 vaccine side effects among HCWs. We included 19 observational studies, most of which were of satisfactory quality, covering 157,981 COVID-19 vaccination events among 96,786 HCWs. Our systematic review provides evidence on both the prevalence and the duration of absenteeism, as well as the associated factors, offering a comprehensive picture of the global burden of absenteeism related to COVID-19 vaccine side effects. Furthermore, we identified several sources of heterogeneity in our analysis, while the risk of a publication bias is considered very low. Although we identified study design as a significant source of between-group heterogeneity, it should be noted that all the included cohort/prospective studies were conducted among mRNA COVID-19 vaccine recipients. We further performed a post-hoc sensitivity analysis among the recipients of either the BNT162b2 mRNA or the mRNA-1273 vaccines and found that study design did not significantly contribute to between-group heterogeneity among the mRNA vaccine recipients, thereby enhancing the reliability of our main analysis’s results.

The prevalence of absenteeism significantly differed after the first and second COVID-19 vaccine doses among HCWs. Since vector vaccines were predominantly used during the early stages of the vaccination campaigns following the suspension of the AstraZeneca vaccine in several countries, we did not conduct a subgroup analysis between vector and mRNA vaccines, as the risk of confounding due to the vaccination dose was considered very high [65].

Some limitations should also be acknowledged. Firstly, despite efforts to identify the potential sources of heterogeneity, it remained high, likely due to not accounting for all the heterogeneity sources simultaneously. This is, however, a common finding in meta-analyses of this proportion [66]. Additionally, although our results were based on studies of satisfactory quality, the majority relied on self-reported questionnaire data for our outcome of interest, which increases the risk of a recall bias, potentially limiting the precision of our findings. Lastly, none of the cross-sectional studies described the characteristics of non-responders, significantly increasing the risk of a selection bias and potentially limiting the generalizability of our findings.

## 5. Conclusions

A non-negligible proportion of the total vaccination events led to at least one day of absenteeism following a COVID-19 vaccination among HCWs. The female participants, the recipients of the second COVID-19 vaccine dose, and the recipients of the mRNA-1273 vaccine reported a higher prevalence of absenteeism related to COVID-19 vaccination. Among the vaccinated HCWs, most of the participants reported 1–2 days of work absence. Our findings suggest that absenteeism related to COVID-19 vaccine side effects may have contributed to the overall increase in absenteeism observed during the COVID-19 pandemic among HCWs. Age, sex, and occupational group should be considered in future vaccination schedules for HCWs to potentially decrease the burden of absenteeism related to vaccine side effects. Future large-scale, prospective studies retrieving data from electronic records could enhance the reliability of our results. As mentioned, factors that were not directly related to immune response and reactogenicity, such as occupational group and income, were found to be associated with absenteeism related to COVID-19 vaccine side effects among HCWs. Further studies could shed light on and further explore the nature of these associations.

## Figures and Tables

**Table 1:** Risk-of-bias assessment and individual studies’ main characteristics.

Study	Country	Population	Risk-of-Bias Assessment
**Cross-sectional studies**			
Bauernfeind et al. (2022) [25]	Germany	A total of 2662 HCWs from Regensburg University Medical Centre and its 10 participating teaching hospitals.	5/10
Chagarlamudi et al. (2021) [27]	USA	A total of 316 HCWs from the department of radiology of a health care institution with 14 hospitals and 57 imaging centers. Only employees whose work required direct in-person duties were included in the study.	2/10
Chrissian et al. (2022) [28]	USA	A total of 2103 HCWs at two large hospital systems (academic and private) in Southern California.	4/10
Cohen et al. (2022) [29]	USA	A total of 861 employees in the departments of Family Medicine, Internal Medicine, Obstetrics and Gynaecology, Paediatrics, and Surgery at Robert Wood Johnson Medical School.	6/10
Kadali et al. (2021) [30]	USA	A total of 432 HCWs from healthcare communities or groups representing various parts of the country.	4/10
Kermer et al. [31]	Germany	A total of 357 HCWs of the Friesland hospitals who voluntarily got vaccinated against COVID-19 with any vaccine/combination.	2/10
Kim et al. (2021) [32]	Korea	A total of 3085 HCWs in three referral teaching hospitals, located in Seoul, Bucheon, and Cheonan, in the Republic of Korea.	5/10
Konu et al. (2021) [33]	Togo	A total of 1639 HCWs who received the first dose of the ChAdOx1 nCoV-19 vaccine during the first phase of the vaccination campaign in Togo.	6/10
Nachtgall et al. (2022) [15]	Germany	A total of 8269 HCWs of the Helios Kliniken Group, a privately owned company with 89 hospitals, ranging from small community structures to university hospitals in Germany.	5/10
Niekrens et al. (2022) [35]	Germany	A total of 2377 employees of the University Hospital of Erlangen vaccinated with the BNT162b2 vaccine.	5/10
Nohl et al. (2022) [36]	Germany	A total of 3905 frontline HCWs from different cities and counties of Germany.	6/10
Parés-Badell et al. (2022) [37]	Spain	A total of 7152 HCWs from the Hospital Universitari Vall d’Hebron were recipients of the COVID-19 mRNA-1273 booster.	5/10
Saita et al. (2022) [39]	Japan	A total of 6419 HCWs a large academic hospital in Japan	4/10
Schianchi et al. (2021) [40]	Italy	A total of 4088 HCWs employed at the Niguarda Hospital.	7/10
**Cohort/Prospective studies**			
Breeher et al. (2022) [26]	USA	HCWs from Mayo Clinic sites: 47,318 received dose 1 and 44,357 received dose 2 of a COVID-19 vaccine.	6/9
Naito et al. (2022) [34]	Japan	A total of 3821 HCWs from seven hospitals within the National Hospital Organization and four hospitals from the Japan Community Health Care Organization were included. HCWs who had completed two BNT162b2 vaccinations and were eligible for the third immunization as a booster dose were enrolled for study participation.	5/9
Reusch et al. (2022) [14]	Germany	Mainly HCWs (n = 1704) from a single tertiary hospital, but HCWs from surrounding hospitals and medical surgeries were also included based on the following criteria: (i) age ≥ 18 years, (ii) written informed consent, (iii) minimum interval of 14 days after first confirmation of SARS-CoV-2 infection by polymerase chain reaction and/or at least one dose of COVID-19 vaccination, regardless of vaccination schedule, and (iv) employment in healthcare sector.	5/9
Risco-Risco et al. (2023) [38]	Spain	A total of 278 HCWs of the HM Sanchinarro, a university teaching hospital in Madrid who received two doses of the BNT162b2 vaccine.	3/9
Wagenhäuser et al. (2022) [41]	Germany	A total of 104 HCWs who were previously administered three COVID-19 vaccine doses and who received a second booster with the monovalent BNT162b2 mRNA vaccine or the bivalent BNT162b2 mRNA original/Omicron BA.4-5 vaccine.	4/9

**Table 2:** Subgroup analysis of the prevalence of at least one day of absenteeism related to COVID-19 vaccine side effects among healthcare workers by i. vaccination dose, ii. study design, iii. region, iv. sex, and v. vaccine type.

Subgroup	Pooled Proportion (95% CI)	Q Statistic *p*-Value	Subgroup I^2^
Study-level sources of heterogeneity			
Study design		0.005	99.7%
Cross-sectional	19% (15–24%)		99.6%
Cohort/Prospective	10% (4–15%)		99.4%
Region		0.006	99.7%
Europe	17% (11–22%)		98.9%
USA	23% (11–35%)		98.9%
Asia	8% (4–12%)		98.9%
Individual-level sources of heterogeneity			
Vaccination		<0.000	99.9%
1st	7% (4–10%)		99.9%
2nd	20% (14–26%)		99.9%
Sex		0.013	97.5%
Female	26% (20–32%)		97.7%
Male	17% (13–21%)		82.8%
Vaccine type		0.009	99.4%
mRNA-1273	23% (16–31%)		99.2%
BNT162b2 mRNA	12% (9–15%)		99.5%

**Table 3:** Duration of absenteeism related to COVID-19 vaccine side effects among health workers who reported a sick leave.

Study	Duration of Absenteeism
Bauernfeind et al. [25]	The median days of sick leave in COVID-19 vaccine recipients without a history of SARS-CoV-2 infection: after first vaccination (n = 239)—Median (IQR): 1 (0–2); after second vaccination (n = 538)—Median (IQR): 1 (0–2).
Chagarlamudi et al. [27]	All the participants who used sick leave took one day (the day following vaccination) of sick leave.
Nachtigall et al. [15]	Of the participants, 76.3% reported a sick leave of 1–2 days, and 23.7% reported 3 or more days.
Naito et al. [34]	After the third dose, 84.6% of the participants took one day of sick leave, 14.8% took two consecutive days, and 0.6% took three consecutive days. The patterns of sick leave between the second and third vaccinations were similar.
Niekrens et al. [35]	After the first dose, the mean days of absence were 1.96. After the second dose, the mean days of absence were 1.74.
Nohl et al. [36]	Days of sick leave after the first vaccination among participants: 38% took one day, 37% took two days, and 25% took three or more days. Days of sick leave after the second vaccination among participants: 47% took one day, 31% took two days, and 22% took three or more days.
Reusch et al. [14]	Average sick days among the participants: 2.43 after the first dose, 1.99 after the second dose, and 1.83 after the third dose.
Saita et al. [39]	Among the recipients of the first dose, 68% needed one day of absence, 8% needed two to three days, and 24% needed four or more days. For the recipients of the second dose, 75.6% needed only one day, 22.4% needed two to three days, and 2% needed four or more days.
Schianchi et al. [40]	The average duration of work absence was 2 days (SD = 1) after both the first and second dose.
Wagenhäuser et al. [41]	The mean duration of work absence in the bivalent-vaccinated group was 2.1 (±3.5) and in the monovalent-vaccinated group was 1.2 (±0.4).

## Data Availability

Detailed methods, results, and additional data are available in the manuscript and the corresponding Supplementary Materials.

## Supplementary Materials

The detailed search strings can be downloaded at https://www.mdpi.com/article/10.3390/vaccines12101196/s1, Data bases search strings.
