# Supplementary material for: The Global Burden of Absenteeism Related to COVID-19 Vaccine Side Effects Among Healthcare Workers: A Systematic Review and Meta-Analysis
Source: Vaccines (Basel). 2024 Oct 19;12(10):1196. doi: 10.3390/vaccines12101196 (PMC11511213; doi:10.3390/vaccines12101196)
Supplement: Supplementary file 1 [file vaccines-12-01196-s001.zip › vaccines-3222933-supplementary.pdf]

Pubmed

("Absenteeism"[MeSH Terms] OR "Presenteeism"[Mesh] OR "Absenteeism"[tiab] OR "Presenteeism"[tiab] OR "workplace absen*"[tiab] OR "workplace presen*"[tiab] OR "work absen*"[tiab] OR "work presen*"[tiab] OR "job absen*"[tiab] OR " job prese*"[tiab] OR ''employee absen*''[tiab] OR ''employee presen*''[tiab] OR ''inability to work''[tiab] OR ''incapacity to work''[tiab] OR ''sick leave''[MeSH Terms] OR ''sick leave''[tiab] OR ''sickness absen*''[tiab]) AND (("COVID-19 Vaccines"[MeSH Terms] OR "COVID-19 vaccin*"[tiab] OR "SARS-CoV-2 vaccin*"[tiab] OR ''coronavirus vaccin*''[tiab]) OR (("Vaccination"[Mesh] OR "Immunization"[Mesh] OR "Immunization Programs"[Mesh] OR "vaccin*"[tiab] OR "immuni*"[tiab] OR "inoculat*"[tiab]) AND ("COVID-19"[Mesh] OR "SARS-CoV-2"[Mesh] OR "COVID-19"[tw] OR "COVID"[tw] OR "SARS-CoV-2"[tw] OR "coronavirus"[tw]))) AND ("Health Personnel"[Mesh] OR "Health Workforce"[Mesh] OR "health personnel"[tiab] OR "healthcare personnel"[tiab] OR "health care personnel"[tiab] OR "health Workforce"[tiab] OR ''healthcare workforce''[tiab] OR ''health care workforce''[tiab] OR "health workers"[tiab] OR "healthcare workers"[tiab] OR "health care workers"[tiab] OR ''health professionals''[tiab] OR ''healthcare professionals''[tiab] OR ''health care professionals''[tiab] OR ''health providers''[tiab] OR ''healthcare providers''[tiab] OR ''health care providers''[tiab])

Embase

('absenteeism'/exp OR 'presenteeism'/exp OR 'absenteeism':ti,ab,kw OR 'presenteeism':ti,ab,kw OR 'workplace absen*':ti,ab,kw OR 'workplace presen*':ti,ab,kw OR 'work absen*':ti,ab,kw OR 'work presen*':ti,ab,kw OR 'job absen*':ti,ab,kw OR 'job prese*':ti,ab,kw OR 'employee absen*':ti,ab,kw OR 'employee presen*':ti,ab,kw OR 'inability to work':ti,ab,kw OR 'incapacity to work':ti,ab,kw OR 'medical leave'/exp OR 'sick leave':ti,ab,kw OR 'sickness absen*':ti,ab,kw) AND (('SARS-CoV-2 vaccine'/exp OR 'covid-19 vaccin*':ti,ab,kw OR 'sars-cov-2 vaccin*':ti,ab,kw OR 'coronavirus vaccin*':ti,ab,kw) OR (('vaccination'/exp OR 'immunization'/exp OR 'preventive health service'/exp OR 'vaccin*':ti,ab,kw OR 'immuni*':ti,ab,kw OR 'inoculat*':ti,ab,kw) AND ('coronavirus disease 2019'/exp OR 'Severe acute respiratory syndrome coronavirus 2'/exp OR 'covid-19':ti,ab,kw,de,dn,df,mn,tn OR 'covid':ti,ab,kw,de,dn,df,mn,tn OR 'sars-cov-2':ti,ab,kw,de,dn,df,mn,tn OR 'coronavirus':ti,ab,kw,de,dn,df,mn,tn))) AND ('health care personnel'/exp OR 'health workforce'/exp OR 'health personnel':ti,ab,kw OR 'healthcare personnel':ti,ab,kw OR 'health care personnel':ti,ab,kw OR 'health workforce':ti,ab,kw OR 'healthcare workforce':ti,ab,kw OR 'health care workforce':ti,ab,kw OR 'health workers':ti,ab,kw OR 'healthcare workers':ti,ab,kw OR 'health care workers':ti,ab,kw OR 'health professionals':ti,ab,kw OR 'healthcare professionals':ti,ab,kw OR 'health care professionals':ti,ab,kw OR 'health providers':ti,ab,kw OR 'healthcare providers':ti,ab,kw OR 'health care providers':ti,ab,kw)

Scopus

((("Absenteeism" OR "Presenteeism") OR ((Incapacity OR inability) PRE/2 (work)) OR (''sick leave'' OR ''sickness absen*'') OR ((work* OR job OR employee) PRE/2 (absen* OR presen*))) AND (("COVID-19 vaccin*" OR "SARS-CoV-2 vaccin*" OR ''coronavirus vaccin*'') OR ((vaccin* OR immuni* OR inoculat*) PRE/2 ("COVID-19" OR "COVID" OR "SARS-CoV-2" OR "coronavirus"))) AND ((health*) PRE/2 (personnel OR workforce OR workers OR professionals OR providers)))

WOS

((("Absenteeism" OR "Presenteeism") OR ((Incapacity OR inability) NEAR/2 (work)) OR (''sick leave'' OR ''sickness absen*'') OR ((work* OR job OR employee) NEAR/2 (absen* OR presen*))) AND (("COVID-19 vaccin*" OR "SARS-CoV-2 vaccin*" OR ''coronavirus vaccin*'') OR ((vaccin* OR immuni* OR inoculat*) NEAR/2 ("COVID-19" OR "COVID" OR "SARS-CoV-2" OR "coronavirus"))) AND ((health*) NEAR/2 (personnel OR workforce OR workers OR professionals OR providers)))
